# Integrated Inflammatory and Nutritional Biomarkers in Metastatic Renal Cell Carcinoma Treated with Second- or Later-Line Nivolumab: A Retrospective Cohort Study

**DOI:** 10.3390/medicina62081520

**Published:** 2026-08-07

**Authors:** Muzaffer Uğraklı, Ülkü Kerimoğlu, Mehmet Zahid Koçak, Talat Aykut, Dilek Çağlayan, Melek Karakurt Eryılmaz, Murat Araz, Mehmet Artaç

**Affiliations:** 1 Department of Medical Oncology, Faculty of Medicine, Necmettin Erbakan University, Konya 42090, Turkey; mehmetzahidkocak@hotmail.com (M.Z.K.); talat_aykut@hotmail.com (T.A.); dilekcaglayan88@gmail.com (D.Ç.); drangelkarakurt@hotmail.com (M.K.E.); zaratarum@yahoo.com (M.A.); mehmetartac@yahoo.com (M.A.); 2 Department of Radiology, Faculty of Medicine, Necmettin Erbakan University, Konya 42090, Turkey; kerimogluulku@yahoo.com

**Keywords:** metastatic renal cell carcinoma, nivolumab, CRP-to-albumin ratio, prognostic nutritional index, psoas muscle index, systemic immune-inflammation index, time-to-treatment failure, Kaplan–Meier analysis, retrospective cohort

## Abstract

*Background and Objectives*: During the 2016–2022 period, tyrosine kinase inhibitor (TKI) monotherapy was the standard first-line option at our center before combination protocols became available. This study evaluates patients experiencing initial TKI failure who subsequently received second- or later-line nivolumab. Because clinical practice lacks low-cost, accessible tools to prognosticate outcomes in this setting, we evaluated whether baseline psoas muscle index (PMI), early on-treatment C-reactive protein (CRP) kinetics, and a specific panel of blood-derived inflammatory/nutritional ratios offer reliable prognostic value. *Materials and methods*: We retrospectively reviewed data from 49 consecutive mRCC patients who progressed on a TKI and received second- or later-line nivolumab monotherapy. Primary endpoints were overall survival (OS; N = 49) and time-to-treatment failure (TTF; N = 48). We utilized Kaplan–Meier and Cox regression models for analysis. Benjamini–Hochberg false discovery rate (FDR) correction (q < 0.05) controlled false positive inflation across two separate testing frameworks: seven log-rank assessments for TTF and the core Cox models. Adjusted models accounted for clinical covariates including International Metastatic RCC Database Consortium (IMDC) risk category, Eastern Cooperative Oncology Group performance status (ECOG PS), and de novo metastatic presentation. We calculated the intraclass correlation coefficient (ICC) to evaluate measurement reproducibility. *Results*: Median follow-up reached 48.4 months (reverse Kaplan–Meier; maximum: 87.8 months), with a cohort-wide median OS of 13.9 months (IQR: 5.8–36.2). A low baseline C-reactive protein-to-albumin ratio (CAR) mapped to a distinct survival benefit, showing a median OS of 32.6 months versus 5.8 months in the high-CAR group (log-rank *p* < 0.001; BH q = 0.003). In adjusted multivariable Cox hazards modeling, both CAR (HR: 1.029; *p* = 0.002) and the prognostic nutritional index (PNI; HR: 0.893; *p* < 0.001) maintained independent prognostic significance. When evaluating the 43 patients with complete 3-month CRP records, we found a significantly higher objective response rate (ORR) in those at or below the cohort-median ratio threshold of 1.16 compared to patients above it (54.5% vs. 23.8%; *p* = 0.039). The overall ITT population registered an ORR of 36.7% (18/49). Conclusions: Low pretreatment CAR and elevated PNI identify mRCC patients showing improved survival profiles in this post-TKI nivolumab subgroup. Additionally, an elevated 3-month CRP ratio correlates with compromised objective response rates and a trend toward shorter TTF, though the TTF endpoint did not clear strict FDR parameters (BH q = 0.098). These retrospective, single-center data serve strictly for signal generation and require independent prospective multi-institutional confirmation before translating to oncology practice.

## 1. Introduction

Globally, renal cell carcinoma (RCC) represents the vast majority of the roughly 338,000 kidney cancer cases diagnosed annually [1]. Clear cell remains the most common histological subtype. Unfortunately, about a quarter to a third of these patients either present with metastatic disease at the outset or face progression later on. Once the disease spreads, achieving a median survival beyond two to three years remains a steep challenge, even with the therapeutic options we have today [1,2]. While immune checkpoint inhibitors have thoroughly reshaped how we treat this disease, predicting right from the start exactly which patients will derive real clinical benefit is still notoriously difficult.

When it comes to second-line treatment, the landmark CheckMate 025 trial established a clear survival advantage for nivolumab over everolimus, posting a median OS of 25.0 versus 19.6 months (HR 0.73; *p* = 0.002) [3]. This data firmly positioned nivolumab as the standard reference agent in regions or clinical scenarios where first-line combination TKI-immunotherapy regimens [4] are not yet part of routine practice. Even so, a significant number of patients see very little benefit from nivolumab. Moreover, no inexpensive, easily scalable biomarker that can tell us who will respond before a single dose is given [3,4].

Beyond tumor biology, the patient’s own physiology matters. Body composition and systemic inflammation are both measurable from CT scans and blood draws that patients already undergo, at no extra cost or burden. Sarcopenia the loss of skeletal muscle mass and function [5] has independently predicted poor outcomes across a range of solid tumor types [5]. The psoas muscle index (PMI) captures this by averaging the bilateral psoas cross-sectional areas at L3 on CT, then dividing by height squared to produce a body-size-adjusted number. Growing evidence links low PMI not just to nutritional depletion but also to reduced immunological reserve, a relationship formally described as the sarco-inflammatory axis [6,7].

Several of these host-related signals can be distilled into simple ratios from the routine full blood count and biochemistry panel [8,9,10]: CAR, NLR, SII, LMR, PLR, SIRI and PNI. All require only standard laboratory results, which makes them inexpensive and broadly applicable as exploratory tools. Less explored is whether inflammatory markers change usefully once treatment starts. A falling CRP after the first nivolumab doses might reflect tumor response; a CRP that climbs could signal progression. The ratio of the 3-month CRP to baseline has shown predictive value in PD-1 blockade trials across several tumor types [11,12] and has recently been examined in first-line mRCC combination regimens [13] and in contemporary real-world checkpoint inhibitor cohorts [14,15]. Whether this same kinetic pattern holds for patients who have already progressed on initial TKI therapy and are receiving nivolumab as a later-line option remains unknown.

This question motivated the present analysis. We therefore conducted a retrospective review of 49 consecutive mRCC patients managed at our tertiary referral center between 2016 and 2022. Every patient in this cohort had experienced disease progression on a prior TKI before moving onto second- or later-line nivolumab monotherapy, with our longest follow-up tracking window extending out to 87.8 months. We set out with a clear set of objectives: to evaluate if baseline PMI_0_, a specific panel of blood-derived inflammatory/nutritional ratios, and early on-treatment CRP kinetics offer genuine prognostic insights. Ultimately, we wanted to see if these accessible variables could be woven together into a practical, composite risk score that merits validation in future prospective trials.

## 2. Materials and Methods

### 2.1. Patient Population and Study Design

This research is a retrospective chart review conducted at the Department of Medical Oncology within the Necmettin Erbakan University Faculty of Medicine in Konya, Turkey. The institutional ethics committee reviewed and approved our study protocol under the designation number 2023/4158 on 20 January 2023. We screened eligible cases between January 2016 and December 2022. This process yielded a cohort of 49 consecutive patients with histologically confirmed mRCC who had experienced objective progression on an initial TKI-based regimen before switching to second- or later-line nivolumab monotherapy. Eligibility required patients to be at least 18 years of age, hold a confirmed mRCC diagnosis, and complete no fewer than two full cycles of nivolumab. Furthermore, the psoas muscle sub-analysis was restricted to individuals who had a contrast-enhanced CT scan within the four weeks preceding their first nivolumab dose, coupled with a verifiable baseline laboratory data profile.

### 2.2. Nivolumab Regimen and Side Effects

Nivolumab was given in accordance with standard oncology practice routines using standard dosing options: 3 mg/kg every two weeks, 240 mg every two weeks, or 480 mg every four weeks. Treatment was maintained continuously until the documentation of objective disease progression, the development of intolerable toxicities, or patient death. We gathered data regarding immune-mediated side effects via retrospective chart extraction, classifying each event severity based on the NCI-CTCAE version 5.0 (National Cancer Institute Common Terminology Criteria for Adverse Eventsversion 5.0; National Cancer Institute, Bethesda, MD, USA; available at: https://ctep.cancer.gov/protocolDevelopment/electronic_applications/ctc.htm, accessed on 15 May 2026) guidelines [16].

### 2.3. Measurement of Psoas Muscle Index

All psoas measurements were performed by one dedicated radiologist (U.K.) on axial CT images at the L3 vertebral level (window 400 HU, level 50 HU) using the institutional PACS (Sectra PACS, Sectra AB, Linköping, Sweden) caliper. Each cross-sectional area was estimated by the ellipsoid method as π/4 × anteroposterior × transverse diameter, a technique previously validated in oncology cohorts [10,17,18], and PMI (cm^2^/m^2^) was calculated as bilateral area/height^2^. Serial assessments were obtained at baseline (PMI_0_), 3 months (PMI_3_) and 6 months (PMI_6_). An independent blinded re-measurement of 20% of datasets confirmed excellent reproducibility (ICC = 0.94; 95% CI: 0.89–0.97; Supplementary Figure S3). All 49 patients fell below published western sex-specific sarcopenia cut-offs (≤3.55 cm^2^/m^2^ in males, ≤2.86 cm^2^/m^2^ in females [5]); the cohort median (1.97 cm^2^/m^2^) was therefore used as the binary threshold. Because this ellipsoid-derived threshold is technique-specific, it should not be compared directly with values from manual or automated segmentation; further methodological details are provided in the Supplementary Materials.

### 2.4. Composite Index Calculation

We derived all peripheral blood indices from the baseline laboratory draws. The definitions used were NLR = ANC/ALC; PLR = platelets/ALC; LMR = ALC/AMC; SII = (platelets × ANC)/ALC; SIRI = (AMC × ANC)/ALC; and CAR = CRP (mg/L)/albumin (g/dL). For the prognostic nutritional index (PNI), we used the following equation based on the absolute lymphocyte count input as ×10^9^/L: PNI = 10 × albumin [g/dL] + 5 × ALC [×10^9^/L]. Lastly, we built an exploratory post hoc risk score ranging from 0 to 2, assigning one point each for a baseline CAR or SII value that sat above the cohort median.

### 2.5. Clinical Response Evaluation

Treating oncologists assessed tumor response per RECIST 1.1 [19] using contrast-enhanced CT every 8–12 weeks. When pseudoprogression was suspected, nivolumab was continued for a further 4–6 weeks in clinically stable patients pending a confirmatory scan. The five patients who were discontinued or died before their first evaluable imaging were counted as non-responders in the ORR denominator to prevent selection bias. OS, measured from the start of nivolumab to death or last follow-up, served as the primary endpoint. TTF the interval from treatment start to discontinuation for any reason, whether progression, toxicity, or patient preference was a secondary exploratory endpoint. Because RECIST 1.1 progression dates were not systematically captured in routine charts, TTF served as a pragmatic, exploratory proxy for clinical benefit rather than a radiographic efficacy measure [19]. One patient with an irreconcilable date entry was omitted from TTF analyses (N = 48).

### 2.6. Statistical Methods

Continuous variables were first tested for normality using the Shapiro–Wilk test; results are presented as medians with interquartile ranges (IQR). Comparisons between groups relied on the Mann–Whitney U test or independent samples *t*-test for continuous variables, and on chi-square or Fisher’s exact tests for categorical data, chosen according to sample size and distribution. Survival outcomes were estimated by the Kaplan–Meier product limit method, with group differences evaluated by the log-rank test.

Cox models were fitted for both univariate and multivariable analyses; Schoenfeld residuals confirmed proportional hazards (global *p* >0.05). All binary cut-offs were fixed at cohort medians before any analysis. Seven TTF log-rank tests and the Cox models formed two predefined families for Benjamini–Hochberg FDR correction (q < 0.05); nominal *p*-values and adjusted q-values are both reported. Optimism-corrected C-statistics came from 1000-iteration bootstrap resampling. Software: IBM SPSS v26.0 (IBM Corp., Armonk, NY, USA) and R v4.3.0. (R Foundation for Statistical Computing, Vienna, Austria; https://www.r-project.org/, accessed on 25 May 2026). OS was the primary endpoint; TTF and ORR were secondary and exploratory. No formal power calculation was carried out this was a retrospective, signal-searching study, and with only 42 OS events even a moderate hazard ratio would be difficult to detect reliably. False positives from parallel testing were partially controlled by BH-FDR; false negatives from small sample size could not be eliminated. As a cross-check of the median threshold approach, ROC-derived (Youden index) cut-offs for CAR (4.63) and PNI fell within 5% of the cohort medians (CAR median 5.00), suggesting our chosen split was not far from a data-optimized alternative. When PMI_0_ disrupted the three-component composite score an unexpected result we constructed a two-component score (CAR + SII) as a purely post hoc exercise (see Section Limitations Methodological Limits and Limits on Inference).

## 3. Results

### 3.1. Patient Flow and Disease Characteristics

All 49 eligible patients entered the OS analysis. A single patient had a chart entry error that made treatment dates irreconcilable, so TTF was analyzed in 48 patients. The 3-month CRP ratio landmark analyses were computed from the full eligible cohort of 49: four patients who died before the 3-month blood draw or lacked day 90 laboratory values were excluded, leaving 45 CRP-ratio-evaluable patients. Of these, 43 had a documented best response and formed the objective response analysis (two lacked a response record), while 44 formed the CRP ratio TTF analysis (the same date error patient was additionally excluded). A STROBE-compliant patient flow diagram summarizing the evaluable numbers and exclusions at each analytical step (OS, N = 49; TTF, N = 48; CRP ratio evaluable, N = 45; CRP ratio ORR, N = 43; CRP ratio TTF, N = 44) is provided as Supplementary Figure S4.

Looking at the baseline demographics, the group included 34 men (69.4%) with a median age of 63 years at diagnosis (IQR: 56–70). In terms of clinical staging and risk, clear cell histology was confirmed in 77.6% of the cohort, 79.6% presented with an ECOG PS of 0, and 73.5% had de novo metastatic disease. Applying the IMDC criteria split our cohort into 14 patients (28.6%) with favorable risk, 20 (40.8%) with intermediate risk, and 15 (30.6%) categorized as poor risk. Nivolumab was administered as a second-line option for 40 patients (81.6%), whereas the remaining nine (18.4%) received it in the third-line setting or beyond. Following nivolumab failure, a further 32.6% of the cohort moved on to subsequent systemic therapies, which were predominantly cabozantinib or axitinib. We have summarized the complete baseline clinical profile in Table 1.

### 3.2. Treatment Results and Side Effects

Within the 49-patient ITT population, we observed an objective response in 18 cases, yielding an ORR of 36.7%. We documented overall disease control in 31 patients (63.3%); conversely, 13 individuals (26.5%) showed immediate progression at their first evaluation, and five (10.2%) either passed away or discontinued therapy before follow-up imaging could be completed. By the time we locked the data, 42 out of 49 patients (85.7%) had died. The cohort-wide median OS stood at 13.9 months (IQR: 5.8–36.2; range 0.4–87.8). Regarding safety profile, we recorded grade 1–2 immune-related adverse events in nine patients (20.5%) and grade 3–4 toxicities in three (6.8%) based on CTCAE v5.0 criteria. Naturally, given the retrospective framework of this study, lower-grade side effects were likely under-reported. Interestingly, patients who developed irAEs showed a numerically longer median OS than those who did not (17.0 vs. 7.4 months). However, this survival gap did not cross the significance line (log-rank *p* = 0.287) and is heavily prone to exposure duration and immortal time bias; we therefore share this purely as a descriptive observation. This difference should not be interpreted as a biological signal. By definition, a patient must survive long enough on treatment to develop and have an irAE recorded, so patients with irAEs are guaranteed a minimum period of survival that patients dying early can never accrue—classic immortal time (guarantee-time) bias. The apparent survival advantage is therefore most parsimoniously explained by differential treatment exposure rather than by any immunological mechanism, and this comparison is reported for descriptive completeness only.

### 3.3. Parameters of Body Composition

**Figure 1:**
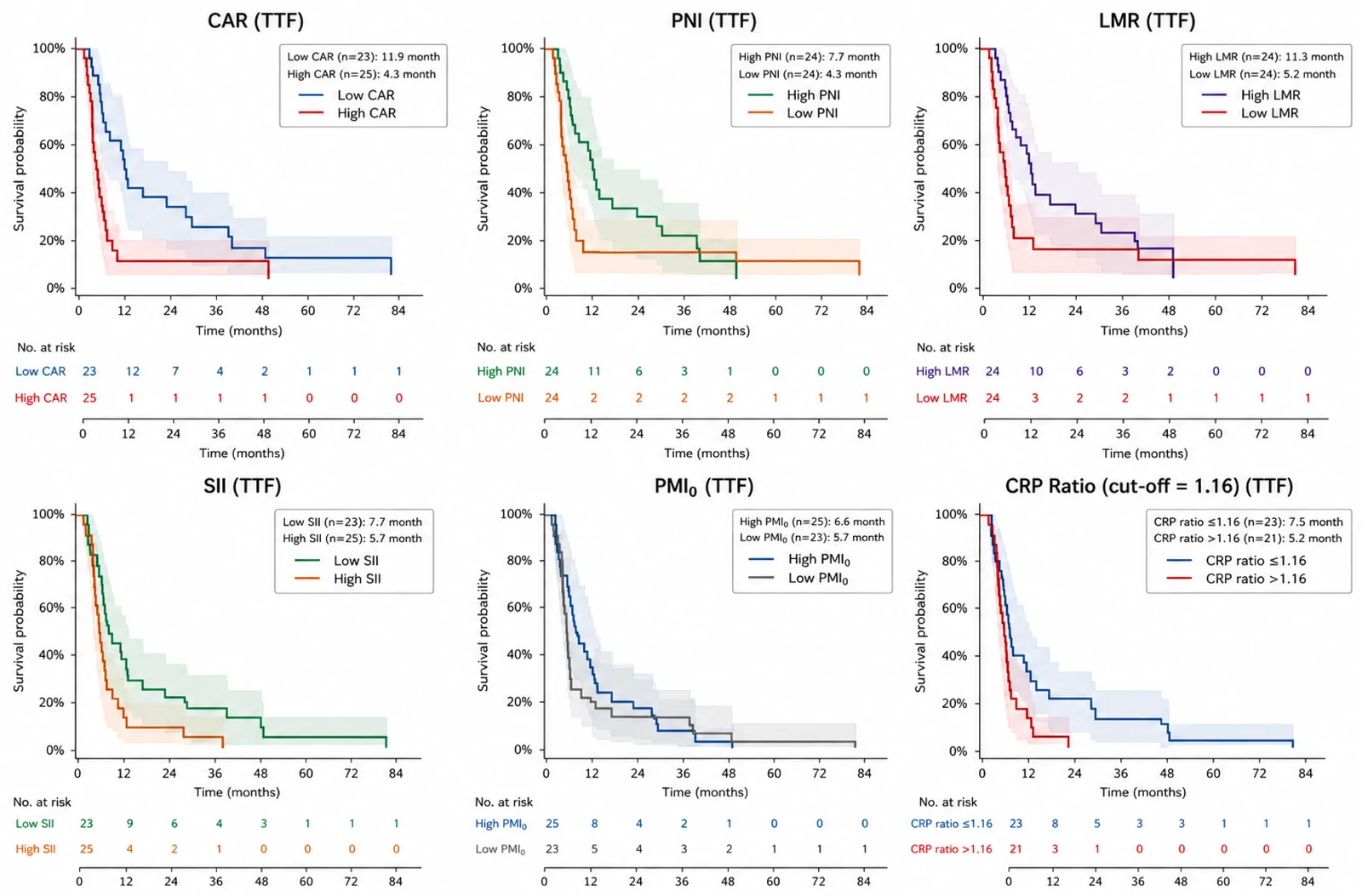
Kaplan–Meier plots of exploratory time-to-treatment failure (TTF) by baseline median thresholds of CAR, PNI, LMR, SII, PMI_0_, and on-treatment CRP ratio (N = 48 overall, CRP ratio panel N = 44). Consecutive numbers at risk are mapped along the timeline.

**Figure 2:**
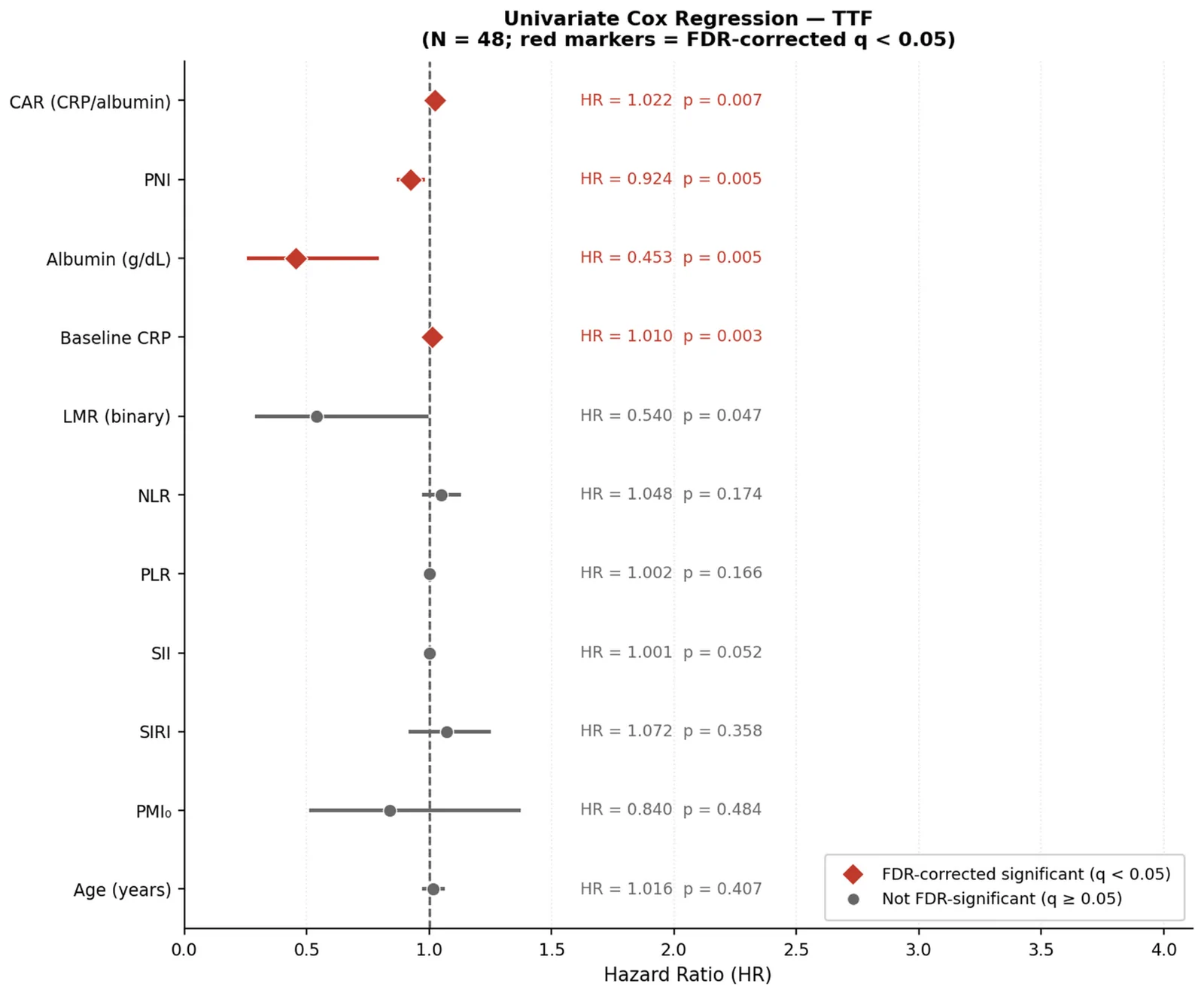
Time-to-treatment failure (TTF; N = 48) univariate Cox hazard ratios (HR) with 95% confidence intervals (CIs). Red markers indicate FDR corrected significance (BH q < 0.05); nominal *p*-values are shown for reference.

Interestingly, pretreatment PMI_0_ failed to distinguish between survival paths, showing no significant separation on Kaplan–Meier curves for OS (*p* = 0.484) or TTF (*p* = 0.771) (Figure 1). This lack of impact was mirrored in our univariate Cox analysis, which confirmed the null hypothesis for both endpoints: OS HR 0.889 (95% CI 0.554–1.428; *p* = 0.627; q = 0.685) and TTF HR 0.840 (95% CI 0.516–1.367; *p* = 0.484) (Table 2). The full set of univariate TTF hazard ratios is shown in Figure 2.” Figure 2 is the forest plot of these hazard ratios. Looking at how muscle mass changed dynamically, psoas mass dropped steadily from baseline to the 3-month and 6-month marks (Wilcoxon *p* < 0.001). However, since the 6-month data points rely solely on survivors who could attend appointments, a classic survivor-selected subgroup, we have restricted these figures to Supplementary Table S1 as a descriptive tracking of muscle depletion over time.

### 3.4. Profiles of Inflammatory and Nutrition Indexes

**Figure 3:**
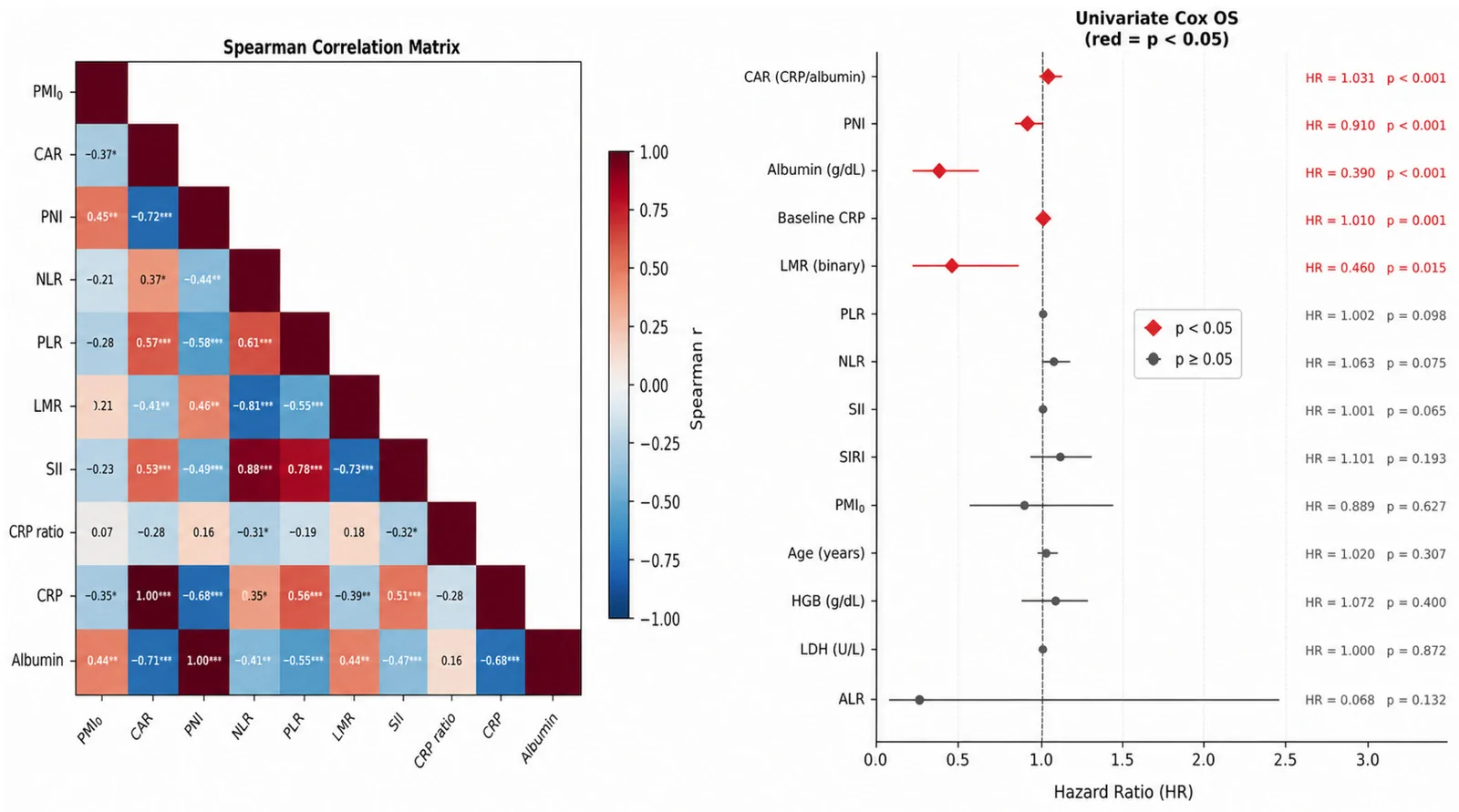
Overall survival was analyzed with a multiparametric Spearman correlation matrix and univariate Cox regression. **Left Panel**: Spearman’s rank correlation matrix of the interactions between baseline metabolic, skeletal and systemic inflammatory indices. **Right Panel**: Forest plot of univariate hazard ratios with corresponding 95% confidence intervals for overall survival (N = 49). * *p* < 0.05; ** *p* < 0.01; *** *p* < 0.001.

Table 1 presents the full distribution of baseline inflammatory and nutritional indices. Our data showed the following cohort medians: CAR 5.00 (IQR: 2.4–19.1), PNI 39.0 (IQR: 33.0–42.0), NLR 3.2 (IQR: 2.3–5.4), PLR 164 (IQR: 116–236), LMR 2.7 (IQR: 1.8–4.0), SII 661.5 (IQR: 416–1159), and a CRP ratio of 1.16 (IQR: 0.46–2.77). Because these markers share common biological pathways, we found that CAR and PNI moved in opposite directions (Spearman r = −0.72; *p* < 0.001), a pattern that was also true for albumin and CRP levels (r = −0.68; *p* < 0.001). For a complete view, the full Spearman correlation matrix and univariate Cox OS forest plot can be reviewed in Figure 3. This strong inverse correlation (r = −0.72) indicates substantial collinearity: CAR, PNI, baseline CRP, and albumin should not be interpreted as independent findings but rather as different mathematical reflections of the same underlying biological axis—systemic inflammation coupled with nutritional depletion and cachexia. Their concordant prognostic direction reinforces this shared biology rather than representing separate, additive prognostic mechanisms.

### 3.5. Analysis of Time-to-Treatment Failure (TTF)

The evaluable cohort generated a median TTF of 8.0 months (IQR: 5.4–12.9; N = 48; CRP ratio analysis: N = 44). Among the baseline markers, CAR emerged as the most powerful tool for separating outcomes: the low-CAR subgroup reached a median TTF of 11.9 months, compared to just 4.3 months for those with high CAR (log-rank *p* < 0.001; Table 3). When we evaluated the dynamic CRP ratio (N = 44), patients tracking at or below the 1.16 median experienced a median TTF of 7.5 months against 5.2 months for those above it (nominal *p* = 0.028). However, strict FDR correction weakened this trend (BH q = 0.098; Table 3), meaning we should view it with caution. Looking at other baseline parameters, only PNI and LMR showed nominal significance (PNI: 7.7 vs. 4.3 months, *p* = 0.042, BH q = 0.098; LMR: 11.3 vs. 5.2 months, *p* = 0.046, BH q = 0.081; Table 3). By contrast, neither SII (7.7 vs. 5.7 months, *p* = 0.069, BH q = 0.097) nor PMI_0_ (6.6 vs. 5.7 months, *p* = 0.771, BH q = 0.771) helped stratify TTF. To clarify what this endpoint captures, we reviewed the documented reason for treatment discontinuation across the 48 evaluable patients. Discontinuation was progression-related in the large majority of cases; among the exceptions, three patients discontinued because of grade 3–4 immune-related toxicity and five patients died early (before the first evaluable scan) without documented radiographic progression, with the small remainder reflecting clinician decision or patient preference. These figures confirm that TTF was progression-dominated in our cohort. Because it also incorporates toxicity and administrative factors; however, TTF should not be equated with RECIST-defined PFS.

We must note that this 1.16 cut-off is just our cohort median. Since it was derived from and applied to the exact same 44 patients, you cannot export it directly to external datasets as a fixed threshold. To cross-check this, we also calculated a Youden index ROC threshold for CAR (4.63). This value approximated the cohort median (5.00) and yielded parallel survival splits (median OS 30.9 vs. 6.2 months; *p* = 0.0005). This gives confidence that the median-based split is a real signal, not a random fluke of the chosen cut-point, though prospective validation with pre-set thresholds is still required.

### 3.6. Kaplan–Meier Survival Analyses

**Figure 4:**
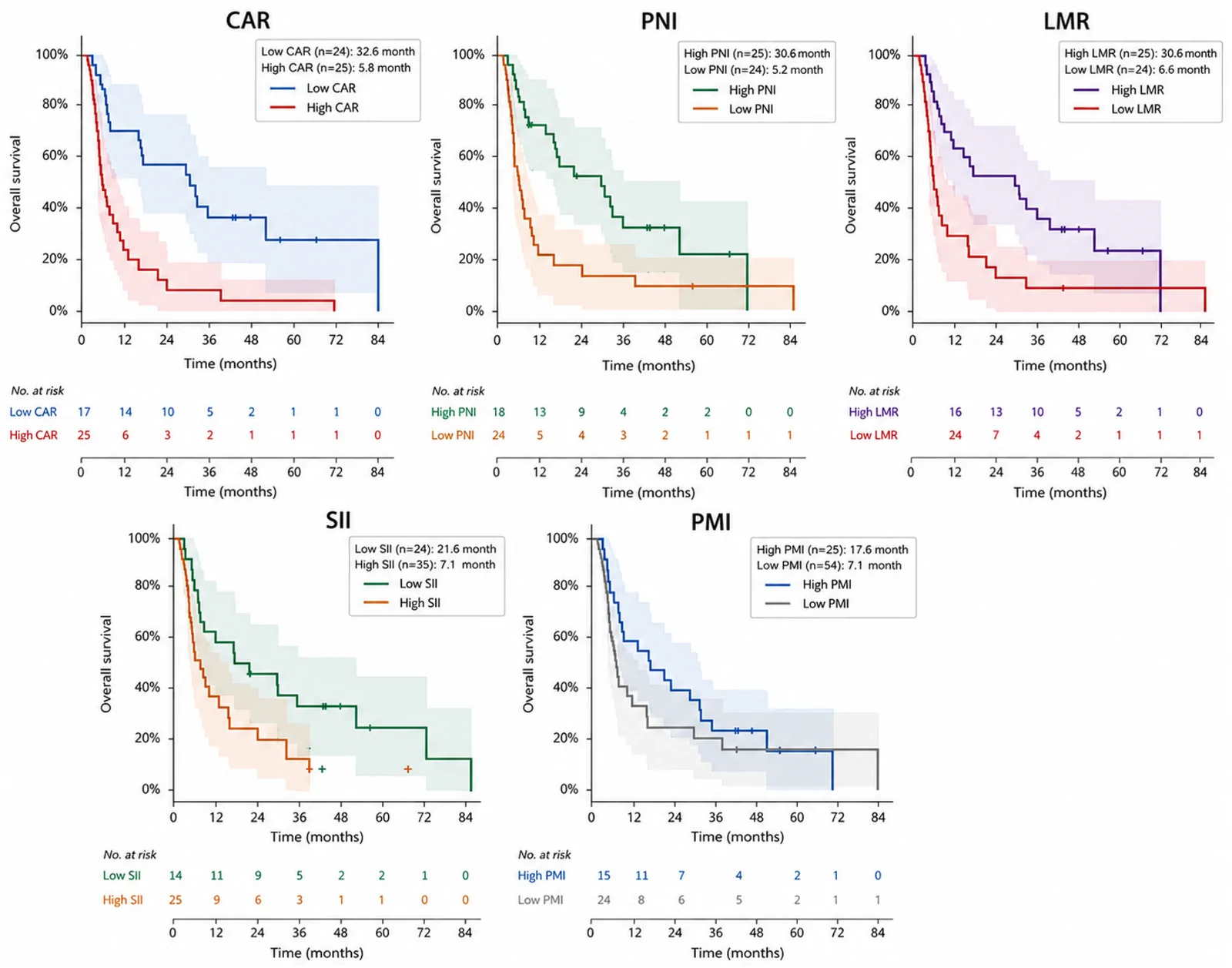
Kaplan–Meier curves for overall survival (OS) according to baseline median cut-off values of CAR, PNI, LMR, SII and PMI_0_. Vertical ticks indicate censored patients. Numbers at risk are shown below the timeline. Log-rank *p*-values and BH-corrected q-values for each stratification are displayed within each panel and are also reported in Table 4; OS was not significantly stratified by PMI_0_ (*p* = 0.627).

CAR yielded the widest separation in overall survival. Low-CAR patients lived a median of 32.6 months compared to 5.8 months in the high-CAR group (log-rank *p* < 0.001; BH q = 0.003). This was tracked over a median follow-up of 48.4 months (reverse Kaplan–Meier; maximum 87.8 months). Following CAR, PNI and LMR served as the next strongest discriminators (PNI: 29.8 vs. 6.2 months, *p* = 0.010, BH q = 0.022; LMR: 30.0 vs. 5.8 months, *p* = 0.013, BH q = 0.024; Figure 4). When focusing solely on patients with clear-cell histology (N = 38) as a sensitivity check, we found the same directional trends for both CAR (*p* < 0.001) and PNI (*p* = 0.012).

**Table 4:** Univariate Cox proportional hazards regression for overall survival (N = 49).

Variable	HR	95% CI	*p*-Value	BH q-Value
CAR (CRP/albumin)	1.031	1.014–1.049	<0.001 ***	<0.001 ***
PNI	0.910	0.872–0.950	<0.001 ***	<0.001 ***
Albumin (g/dL)	0.390	0.247–0.616	<0.001 ***	<0.001 ***
Baseline CRP (mg/L)	1.010	1.004–1.016	0.001 **	0.004 **
LMR (binary)	0.460	0.246–0.862	0.015 *	0.042 *
NLR	1.063	0.993–1.137	0.075	0.150
PLR	1.002	0.999–1.005	0.098	0.172
SII	1.001	1.000–1.002	0.065	0.150
SIRI	1.101	0.952–1.275	0.193	0.270
ALR	0.068	0.002–2.241	0.132	0.205
PMI_0_ (cm^2^/m^2^)	0.889	0.554–1.428	0.627	0.675
HGB (g/dL)	1.072	0.912–1.260	0.400	0.467
LDH (U/L)	1.000	0.997–1.003	0.872	0.872
Age (years)	1.020	0.982–1.060	0.307	0.391

HR: hazard ratio; CI: confidence interval; CAR: CRP-to-albumin ratio; PNI: prognostic nutritional index; LMR: lymphocyte-to-monocyte ratio; SII: systemic immune-inflammation index; PMI: psoas muscle index. Significance: * *p* < 0.05; ** *p* < 0.01; *** *p* < 0.001. Note: CAR is calculated using CRP in mg/L and albumin in g/dL (median cohort CAR = 5.00). Multiply or divide by 10 when comparing with cohorts using CRP in mg/dL (equivalent threshold: 0.50). ALR (albumin-to-LDH ratio) is reported for completeness, but its extremely wide confidence interval (HR 0.068; 95% CI 0.002–2.241) reflects scale and instability; it was not retained in any multivariable model and should not be interpreted as a reliable prognostic estimate; HGB: hemoglobin; LDH: lactate dehydrogenase; ALR: albumin-to-LDH ratio.

### 3.7. Multivariable Cox Proportional Hazards Model

Five baseline indices cleared the BH correction hurdle in univariate Cox screening: CAR (HR: 1.031; *p* < 0.001; q < 0.001), PNI (HR: 0.910; *p* < 0.001; q < 0.001), albumin (HR: 0.390; *p* < 0.001; q < 0.001), baseline CRP (HR: 1.010; *p* = 0.001; q = 0.004), and LMR (HR: 0.460; *p* = 0.015; q = 0.042). We could not enter CAR and PNI into the same multivariable equation because their Spearman correlation was −0.72 (*p* < 0.001). This level of collinearity makes coefficient estimates unstable in a small sample. Therefore, we evaluated them in two separate adjusted models. After controlling for classical covariates namely IMDC risk score, ECOG PS, and de novo metastatic status both CAR (HR: 1.029, 95% CI: 1.011–1.048; *p* = 0.002) and PNI (HR: 0.893, 95% CI: 0.843–0.946; *p* < 0.001) retained independent prognostic value (Table 5).

Internal bootstrap validation across 1000 resamples backed these findings: 99.8% of the runs showed an HR > 1.0 for CAR, and 100.0% showed an HR < 1.0 for PNI. Optimism-corrected C-statistics came out to 0.651 and 0.644, respectively. None of our three clinical adjustment covariates reached statistical significance (all *p* > 0.24). This lack of effect likely stems from how uniform our cohort was: 79.6% had a PS of 0 and the IMDC categories were tightly packed rather than faulty model design. In fact, reshaping IMDC into a binary variable did not shift the CAR or PNI hazard ratios in any meaningful way. It is also worth noting that CAR and PNI share biological ground with SII and LMR; thus, Models A and B should be viewed as exploratory sensitivity checks rather than a single definitive calculation, and their hazard ratios do not represent completely isolated effect sizes. Finally, the albumin-to-LDH ratio returned an erratic, uninterpretable estimate (HR 0.068; 95% CI: 0.002–2.241) due to near-perfect data separation, forcing us to exclude it from all multivariable testing.

### 3.8. Post Hoc Composite Inflammatory Score

When we attempted to merge PMI_0_, SII, and CAR into a unified three-part scoring system, the predictable step-by-step survival gradient collapsed. Paradoxically, patients with a score of 1 outlived those with a score of 0. We identified PMI_0_ as the source of this distortion; it failed to bring any independent prognostic value to the table (Cox OS *p* = 0.627) and instead introduced noise into the composite calculation. Stripping PMI_0_ out left us with a much cleaner two-part tool based on CAR + SII. In this revised setup, a score of 0 (both markers low; *n* = 17) captured a stellar median OS of 30.9 months. A score of 1 (one elevated marker; *n =* 14) dropped to 11.4 months, and a score of 2 (both markers high; *n =* 18) plummeted to 5.2 months (*p* < 0.001 when comparing score 0 directly to score 2; BH q < 0.001). While this secondary adjustment polished the survival gradient, we must acknowledge that this refinement was driven by looking directly at our own results. Consequently, the stark differences reported here are almost certainly overly optimistic, making prospective testing in a fresh patient group mandatory before this index can be taken seriously.

### 3.9. CRP Ratio and Early Response

**Figure 5:**
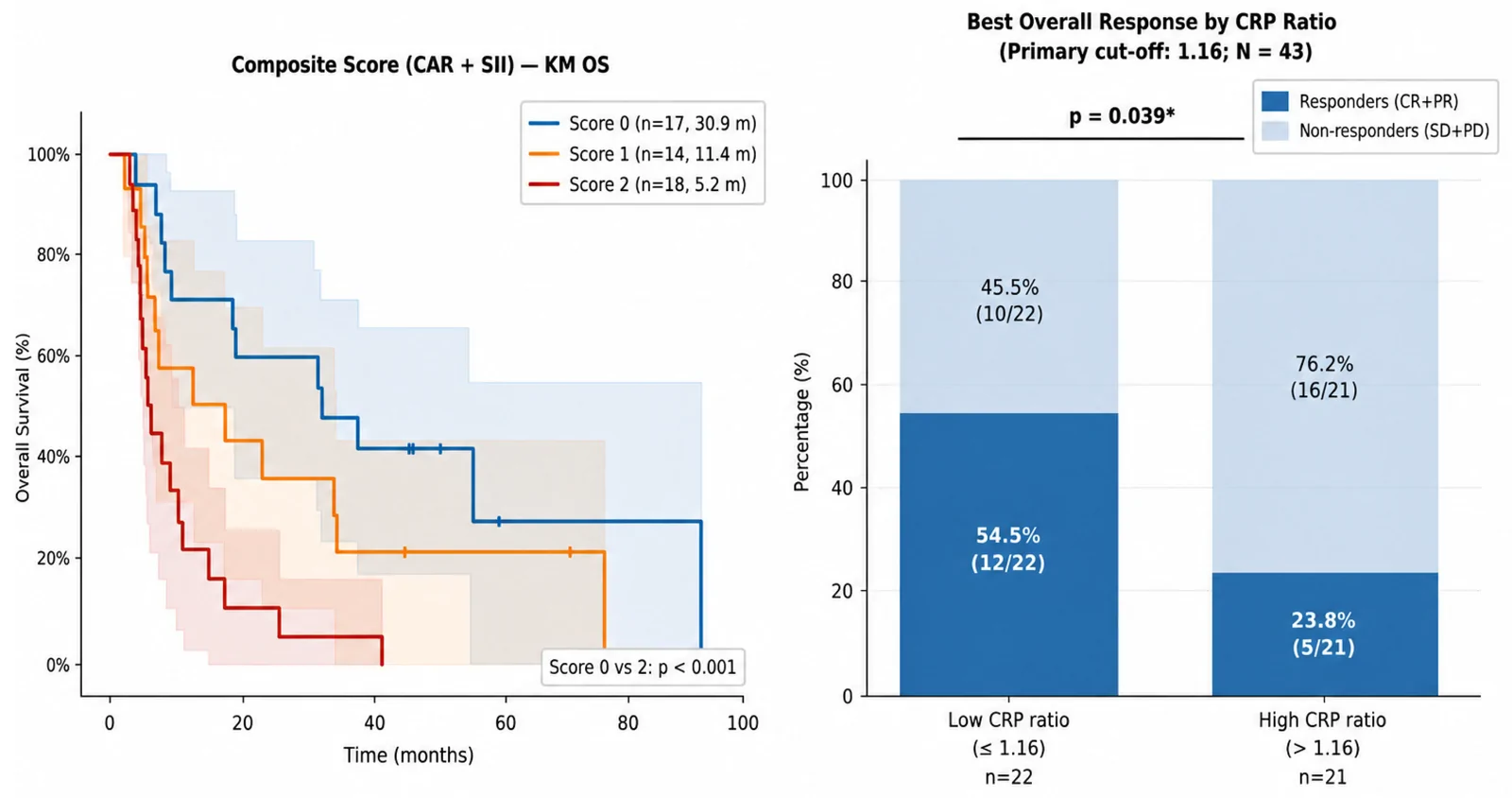
Clinical response distribution and post hoc survival analysis of the scores. **Left Panel**: Kaplan–Meier overall survival curves by the exploratory two-component post hoc score (Scores 0, 1 and 2). **Right Panel**: Distribution of responders (CR + PR) and non-responders (SD + PD) according to cohort-median 3-month on-treatment CRP ratio threshold (≤1.16 vs. >1.16; N = 43). “*” symbol indicates a statistically significant difference.

The 3-month CRP ratio was analyzed as an exploratory, on-treatment marker; the dichotomizing threshold was pre-specified as the cohort median (1.16) rather than data-optimized. Using this cut-off, patients with a low 3-month CRP ratio (*n =* 22; N = 43 evaluable) had an ORR of 54.5%, compared with 23.8% in those above the cut-off (*n =* 21; Chi-square *p* = 0.039). This is displayed in the right panel of Figure 5. The TTF comparison used N = 44 (one additional patient had TTF data despite lacking a recorded response).

### 3.10. Correlations Between PMI_0_ and Inflammatory Markers

We found that higher baseline PMI_0_ systematically aligned with a healthier systemic profile, tracking with lower CAR (r = −0.34; *p* = 0.016), lower PLR (r = −0.30; *p* = 0.036), and lower CRP levels (r = −0.33; *p* = 0.021). Concurrently, it showed positive links with albumin (r = 0.43; *p* = 0.002) and PNI scores (r = 0.44; *p* = 0.002). We have mapped this full baseline correlation web in the left panel of Figure 3.

## 4. Discussion

In this retrospective cohort of second- or later-line nivolumab-treated mRCC, two routinely available baseline markers—the CRP-to-albumin ratio (CAR) and the prognostic nutritional index (PNI)—emerged as the most consistent prognostic signals for overall survival, while an early on-treatment rise in the CRP ratio tracked poorer response. Second- or later-line nivolumab after TKI failure sits in a clinical niche that has attracted relatively little dedicated biomarker research. Most published work focuses on either first-line TKI treatment [9] or combination immunotherapy regimens [3,4]; patients who moved from TKI to single-agent nivolumab are underrepresented. We used a consecutive real-world series to explore this gap. The results are preliminary and not practice-changing on their own; they are intended to generate hypotheses for future prospective testing.

CAR condenses two interconnected adverse processes into one number: active systemic inflammation and protein–energy deficit. Either of these, and certainly their combination, can erode cytotoxic T-cell function and tilt the tumor microenvironment towards immune tolerance [20]. Published meta-analyses in advanced RCC have consistently associated elevated pretreatment CAR with poorer survival [21,22], in line with what we observed here. A practical note on units: we expressed CRP in mg/L and albumin in g/dL, yielding a cohort median CAR of 5.00. Studies that report CRP in mg/dL, including Hara et al. [23], Qi et al. [21], and Li et al. [22], will show thresholds roughly ten-fold lower a unit difference, not a biological one; the directional finding (higher CAR, worse outcome) holds across all conventions. These observations are consistent with contemporary evidence: a recent multicenter analysis confirmed that baseline serum CRP predicts overall survival in both clear-cell and non-clear-cell RCC treated with checkpoint blockade [15], and biomarker work from CheckMate 214 has similarly implicated the inflammatory state of the tumor microenvironment in ICI outcomes [24]. Peripheral immune parameters such as the absolute lymphocyte count have likewise retained prognostic value in recent real-world ICI cohorts [14].

PNI moved in the same direction. Both PNI and albumin reached *p* < 0.001 on univariate Cox analysis, a finding consistent with a meta-analysis spanning checkpoint inhibitor trials across solid tumor types [25]. Low albumin in this setting probably reflects several overlapping problems at once impaired hepatic synthesis, a sustained acute-phase response, and inadequate nutritional intake each of which can independently limit the expansion of effector T cells after PD-1 blockade [20]. Baseline LMR also separated OS meaningfully (30.0 vs. 5.8 months; *p* = 0.015). A similar pattern was seen by the Meet-URO 15 group [8] in a larger mRCC nivolumab cohort, which gives our exploratory observation at least some external plausibility despite the small sample.

**Figure 6:**
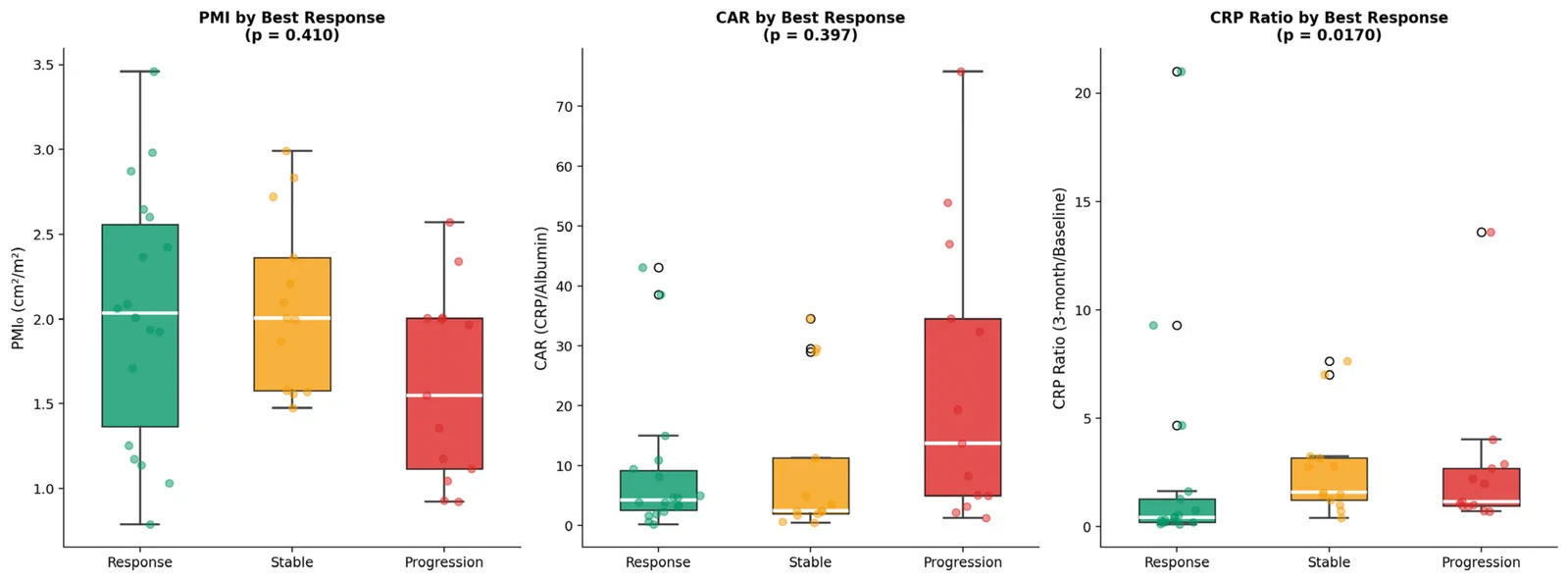
Baseline marker distributions by clinical response tier. Boxplots across patients of single values of baseline PMI_{0}, baseline CAR and 3-month dynamic CRP ratio, across the documented best objective response categories. *p*-values were determined by the Kruskal–Wallis test for the comparison of three groups. For CAR, a pairwise comparison (responders vs. progressors; Mann–Whitney U) showed a tendency to higher CAR in progressors (median 13.8 vs. 4.3; *p* = 0.055).

The 3-month to baseline CRP ratio showed a clinically meaningful association with response. At our prespecified cut-off of 1.16 (cohort median), ORR was 54.5% in the low-ratio group versus 23.8% in the high-ratio group (*p* = 0.039) (Figure 6); anchoring the comparison to a predetermined threshold makes this the least data-driven of our response analyses. Rising on-treatment CRP has been flagged as a marker of inadequate PD-1 blockade in lung cancer [11], urological cancers treated with PD-1/PD-L1 inhibitors [12], and first-line mRCC combination regimens [13]; seeing a directionally similar finding here in second- or later-line monotherapy adds a small piece to that picture. Two limitations must be kept in mind: the 1.16 threshold was derived from and applied to the same cohort, so it defines equal halves of this dataset by construction and will not automatically transfer elsewhere; and the TTF association with this threshold did not survive FDR correction (q = 0.098). A Youden index ROC threshold for CAR (4.63) tracked closely with the median (5.00) and produced similar survival separation (median OS 30.9 vs. 6.2 months; *p* = 0.0005). This is mildly reassuring for the stability of our split. Nonetheless, prospective testing with a fixed, pre-set threshold in an independent cohort is required to confirm whether this signal holds.

All patients in our cohort fell below Western sex-specific sarcopenia cut-offs [5], a sarcopenia rate higher than the 60–90% reported in comparable mRCC immunotherapy datasets [6,7,26]. We think this gap is largely methodological: the ellipsoid caliper approach we used systematically underestimates absolute psoas area compared with manual segmentation, pulling all values downward. PMI_0_ itself was not prognostic for OS or TTF when tested alone, yet its correlations with the inflammatory panel are worth noting. It tracked inversely with CAR (r = −0.34; *p* = 0.016), baseline CRP (r = −0.33; *p* = 0.021), and PLR (r = −0.30; *p* = 0.036), and positively with albumin (r = 0.43; *p* = 0.002) and PNI (r = 0.44; *p* = 0.002; Figure 3). Taken together, these correlations fit the sarco-inflammatory axis concept [27,28]: muscle mass appears to be a biological mirror of the patient’s nutritional and inflammatory state, even when it does not independently separate survival groups. Our cohort median (1.97 cm^2^/m^2^) sat below Asian-derived thresholds too (e.g., Ueki et al. [10]), so cross-study comparison of absolute PMI values is unreliable in this setting; within-cohort median ranking was the only appropriate stratification strategy, and Turkish-specific reference values remain to be developed. Importantly, this uniformity has a direct analytical consequence: because every patient was sarcopenic by published thresholds and PMI_0_ values clustered within a narrow band (median 1.97 cm^2^/m^2^; IQR 1.57–2.49), the cohort offered restricted variability against which to detect a prognostic gradient. Our null PMI_0_ result should therefore not be read as evidence that PMI lacks prognostic value in mRCC; rather, this cohort was unable to test that hypothesis adequately, and PMI_0_ remains worth evaluating in populations with a wider spread of muscle mass.

The three-component score (PMI_0_ + CAR + SII) did not behave as expected: score-1 patients outlived score-0 patients, reversing the intended gradient. PMI_0_ was the most plausible explanation: it carried no Cox-defined OS weight (*p* = 0.627) yet was enough to destabilize the composite. Removing it gave a two-component model (CAR + SII) with median OS of 30.9, 11.4, and 5.2 months across scores 0, 1, and 2. SII was retained in preference to PNI or LMR for two reasons. First, PNI shares albumin with CAR (Spearman r = −0.72), so a CAR + PNI score would largely duplicate the same albumin-driven axis, whereas SII is derived from platelet, neutrophil, and lymphocyte counts and therefore contributes a partly independent cellular inflammatory dimension. Second, LMR was itself strongly correlated with SII (r = −0.73), making the two largely interchangeable. Given its post hoc, data-driven derivation, this composite score is presented solely as an exploratory, hypothesis-generating observation: the separation seen almost certainly overstates the true effect, the *p*-value does not correct for that selection, and pairwise contrasts should carry a Bonferroni-type penalty. It is not intended as a validated risk stratification instrument, and Table 6 and Figure 5 should be read in that exploratory light rather than as evidence of clinical utility. The score should not inform practice without prospective external validation in an adequately powered independent cohort.

### Limitations Methodological Limits and Limits on Inference

The most important caveat is sample size. Forty-nine patients with 42 OS events is a small dataset; effect estimates carry wide confidence intervals, and multivariable models are at genuine risk of overfitting even when the events-per-variable ratio (~10.5) clears conventional entry thresholds. What we report should be read as directional signals particular to this cohort, not as validated prognostic tools, and each finding will need replication in a prospective, adequately powered, multicenter study before it can inform clinical decisions. Specifically, with 42 events across four covariates per model (EPV ≈ 10.5), the margin above conventional thresholds is thin and raises a real risk of over-adjustment; the multivariable estimates should therefore be read as adjusted, exploratory directional signals rather than independently validated prognostic factors.

Testing multiple indices in parallel raises the false positive rate regardless of statistical correction, and BH-FDR only partially addresses this. As a single-center retrospective series, our cohort is also subject to selection bias. In particular, 79.6% of patients had ECOG PS 0 at nivolumab initiation—higher than in unselected mRCC populations and reflecting our tertiary referral pattern toward fitter patients (broadly comparable to the CheckMate 025 Karnofsky ≥ 90% rate of about 72%). Biomarker thresholds derived from a predominantly PS-0 population may not transfer well to patients with worse baseline function, and effect sizes could be inflated relative to a more representative sample. That said, a post hoc comparison of baseline CAR (median 4.63 vs. 8.75; Mann–Whitney *p* = 0.552) and PNI (4.11 vs. 3.61; *p* = 0.140) between PS-0 (*n =* 39) and PS-1 (*n =* 10) patients showed no statistically significant difference, which at least argues that the PS imbalance did not obviously distort the thresholds used for stratification.

Psoas areas came from the ellipsoid caliper method on our PACS workstation rather than dedicated segmentation software. This underestimates absolute area, but the ICC of 0.94 confirms that patient-to-patient ranking within the cohort is stable and that the median-based binary split is biologically defensible. RECIST 1.1 progression dates were not recorded systematically in our charts, so treatment discontinuation was used to define TTF. TTF captures a mix of progression, toxicity, and patient or clinician decisions; it is thus a pragmatic endpoint for a real-world dataset, but it is a noisy surrogate for RECIST-defined PFS and should not be treated as a radiographic efficacy measure. The substitution of PFS by TTF is therefore a genuine limitation, and the TTF-based estimates should be interpreted with corresponding caution. The 6-month psoas data are survival time-biased in the most straightforward way—only patients still alive and in follow-up contributed—and are included as a descriptive longitudinal picture only. The CRP ratio cut-off of 1.16 is the cohort median, derived and tested in the same patients; it splits the cohort into equal halves by arithmetic necessity and is not transferable to other centers without validation, and its TTF signal did not survive FDR correction (q = 0.098). Importantly, because the 3-month CRP ratio can only be computed in patients who survived and remained on treatment to the 3-month landmark, the four patients who died early or lacked day 90 laboratory values were necessarily excluded. This introduces immortal time (guarantee-time) bias that selects for patients healthy enough to reach 3 months and may inflate both the ORR and TTF estimates derived from the CRP ratio subgroup; these results should therefore be read as on-treatment, landmark-conditioned signals rather than baseline prognostic findings. In addition, CRP is an acute-phase reactant, and the 3-month value may be confounded by intercurrent infection, corticosteroid exposure (including steroids given for immune-related adverse events), active irAEs, or intercurrent comorbidity; these events were not systematically captured in our retrospective records and could not be adjusted for, which further limits the causal interpretation of the CRP ratio signal. Finally, post-nivolumab treatment with cabozantinib or axitinib (received by 32.6% of patients) was not modelled as a time-varying covariate and may have influenced the observed OS differences.

## 5. Conclusions

In 49 mRCC patients treated with second- or later-line nivolumab after TKI failure, pretreatment CAR and PNI were the most consistent prognostic markers, holding up in adjusted Cox models. Neither index is new to oncology, but seeing them behave this way specifically in the post-TKI nivolumab context, a setting with relatively little dedicated data, is worth documenting. A rising 3-month CRP ratio was linked to lower response rates and a nominally shorter TTF, though the TTF finding did not pass FDR correction (BH q = 0.098) and cannot be treated as established. A data-driven post hoc two-component score (CAR + SII) separated patients into three survival groups, but the selection of components after the fact almost certainly inflates the apparent effect. Taken together, these are observations from one center; prospective multicenter studies with predefined thresholds are the only meaningful next step. We emphasize that this composite score was generated post hoc after inspecting which components reached nominal significance; it therefore carries a substantial risk of type I error, holds no clinical validity in the absence of external validation, and is presented solely as a hypothesis-generating observation for future prospective testing.

## Figures and Tables

**Table 1:** Baseline patient and disease characteristics, laboratory parameters, and composite indices (N = 49).

Patient and Clinical Characteristics	Value	Laboratory Parameters and Composite Indices	Value
Biochemistry			
Age, median (IQR), years	63 (56–70)	CRP, mg/L	19.0 (10.0–60.0)
Age range, years	38–84	Albumin, g/dL	4.0 (3.5–4.3)
Sex, *n* (%)		LDH, U/L	197 (156–291)
Male	34 (69.4)	Total protein, g/dL	7.2 (6.8–7.6)
Female	15 (30.6)	Bilirubin, mg/dL	0.5 (0.3–0.7)
ECOG PS, *n* (%)		Composite Indices	
0	39 (79.6)	NLR	3.2 (2.3–5.4)
1	9 (18.4)	PLR	164 (116–236)
2	1 (2.0)	LMR	2.7 (1.8–4.0)
IMDC risk, *n* (%)		SII	661.5 (416–1159)
Favorable	14 (28.6)	SIRI	1.2 (0.7–2.4)
Intermediate	20 (40.8)	CAR	5.0 (2.4–19.1)
Poor	15 (30.6)	PNI	39.0 (33.0–42.0)
Histology, *n* (%)		ALR	0.02 (0.01–0.03)
Clear cell	38 (77.6)	PMI_0_, cm^2^/m^2^	1.97 (1.57–2.49)
Non-clear cell	11 (22.4)	CRP ratio (3mo/baseline)	1.16 (0.46–2.77)
De novo metastatic, *n* (%)	36 (73.5)	Survival Outcomes	
Prior nephrectomy, *n* (%)	33 (67.3)	Median OS, months (IQR)	13.9 (5.8–36.2)
Prior TKI, *n* (%)		OS range, months	0.4–87.8
Sunitinib	29 (59.2)	Median TTF, months (IQR)	8.0 (5.4–12.9)
Pazopanib	12 (24.5)	OS events, *n* (%)	42 (85.7)
Other	8 (16.3)	TTF events, *n* (%)	47 (97.9)
No. of metastatic sites, median	2 (1–4)		
Lung metastasis, *n* (%)	29 (59.2)		

Abbreviations: ECOG PS: Eastern Cooperative Oncology Group performance status; IMDC: International Metastatic RCC Database Consortium; IQR: interquartile range; NLR: neutrophil-to-lymphocyte ratio; PLR: platelet-to-lymphocyte ratio; LMR: lymphocyte-to-monocyte ratio; SII: systemic immune-inflammation index; SIRI: systemic inflammation response index; CAR: CRP-to-albumin ratio; PNI: prognostic nutritional index; PMI_0_: baseline psoas muscle index; TKI: tyrosine kinase inhibitor. Note: CAR is calculated using CRP in mg/L and albumin in g/dL (cohort median CAR = 5.00). When comparing with cohorts using CRP in mg/dL, multiply or divide thresholds by 10 (equivalent: 0.50).

**Table 2:** Univariate Cox proportional hazards regression for time-to-treatment failure (TTF; exploratory surrogate endpoint) (N = 48).

Variable	HR	95% CI	*p*-Value	BH q-Value
CAR (CRP/albumin)	1.022	1.006–1.038	0.007 **	0.019 *
PNI	0.924	0.874–0.976	0.005 **	0.018 *
Albumin (g/dL)	0.453	0.261–0.785	0.005 **	0.018 *
Baseline CRP (mg/L)	1.010	1.003–1.017	0.003 **	0.018 *
LMR (binary)	0.540	0.295–0.991	0.047 *	0.095
NLR	1.048	0.979–1.122	0.174	0.239
PLR	1.002	0.999–1.005	0.166	0.239
SII	1.001	1.000–1.002	0.052	0.095
SIRI	1.072	0.924–1.245	0.358	0.438
PMI_0_ (cm^2^/m^2^)	0.840	0.516–1.367	0.484	0.484
Age (years)	1.016	0.978–1.055	0.407	0.448

HR: hazard ratio; CI: confidence interval; CAR: CRP-to-albumin ratio; PNI: prognostic nutritional index; LMR: lymphocyte-to-monocyte ratio; SII: systemic immune-inflammation index; PMI: psoas muscle index. Significance: * *p* < 0.05; ** *p* < 0.01. Asterisks next to *p*-values denote nominal significance (*p* < 0.05); FDR-adjusted significance should be interpreted according to BH q-values. Only variables with q < 0.05 achieve FDR-corrected significance.

**Table 3:** Kaplan–Meier time-to-treatment failure (TTF) analysis by prognostic index (N = 48). All TTF-based inferences are exploratory.

Variable	*n* (G1)	Median TTF G1 (Months)	*n* (G2)	Median TTF G2 (Months)	*p*-Value/BH q-Value
CAR (low vs. high)	23	11.9	25	4.3	<0.001/<0.001 *
PNI (high vs. low)	24	7.7	24	4.3	0.042/0.098
LMR (high vs. low)	24	11.3	24	5.2	0.046/0.081
SII (low vs. high)	23	7.7	25	5.7	0.069/0.097
CRP ratio ≤ 1.16 vs. >1.16 (N = 44)	23	7.5	21	5.2	0.028/0.098
PMI_0_ (high vs. low)	25	6.6	23	5.7	0.771/0.771
NLR (low vs. high)	24	10.2	24	6.0	0.128/0.149

G1: favorable group; G2, unfavorable group; TTF: time-to-treatment failure; CAR: CRP-to-albumin ratio; PNI: prognostic nutritional index; LMR: lymphocyte-to-monocyte ratio; SII: systemic immune-inflammation index; PMI_0_: psoas muscle index. CRP ratio: N = 44 for TTF analysis; N = 43 for objective response analysis (one patient had TTF data but no evaluable response record). BH q-values reflect Benjamini–Hochberg FDR correction applied within the Kaplan–Meier TTF comparison family (7 tests). An asterisk (*) denotes FDR-corrected significance (BH q < 0.05); nominal *p* < 0.05 without FDR correction is not starred.

**Table 5:** Multivariable Cox proportional hazards regression for overall survival—Model A (CAR) and Model B (PNI), adjusted for IMDC risk score, ECOG performance status, and de novo metastatic status (N = 49).

Variable	HR	95% CI	*p*-Value
**Model A (CAR)**			
CAR (CRP-to-albumin ratio)	1.029	1.011–1.048	0.002 **
IMDC risk score (0–2)	1.189	0.753–1.878	0.458
ECOG PS (0–2)	1.461	0.768–2.780	0.248
De novo metastatic	0.914	0.473–1.766	0.791
**Model B (PNI)**			
PNI	0.893	0.843–0.946	<0.001 ***
IMDC risk score (0–2)	1.148	0.718–1.835	0.565
ECOG PS (0–2)	1.383	0.710–2.692	0.339
De novo metastatic	0.862	0.442–1.683	0.662

HR: hazard ratio; CI: confidence interval; CAR: CRP-to-albumin ratio; PNI: prognostic nutritional index; IMDC: International Metastatic RCC Database Consortium; ECOG: Eastern Cooperative Oncology Group.Significance: ** *p* < 0.01; *** *p* < 0.001.

**Table 6:** Revised two-component inflammatory risk score (CAR + SII) and median overall survival by score category (N = 49).

Score	Risk Components	*n* (%)	Median OS (Months)
0	Low CAR + Low SII (no adverse components)	17 (34.7%)	30.9
1	High CAR OR High SII (one adverse component)	14 (28.6%)	11.4
2	High CAR + High SII (both adverse components)	18 (36.7%)	5.2

CAR: CRP-to-albumin ratio; SII: systemic immune-inflammation index; OS: overall survival. Score thresholds (cohort medians): SII ≥ 661.5 (high); CAR ≥ 5.00 (high). Score 0 vs. 2: log-rank *p* < 0.001.

## Data Availability

The data that support the findings of this study are available from the corresponding author upon reasonable request.

## Supplementary Materials

The following supporting information can be downloaded at https://www.mdpi.com/article/10.3390/medicina62081520/s1, The STROBE checklist for observational studies is completed.
