# Supplementary material for: Integrated Inflammatory and Nutritional Biomarkers in Metastatic Renal Cell Carcinoma Treated with Second- or Later-Line Nivolumab: A Retrospective Cohort Study
Source: Medicina (Kaunas). 2026 Aug 7;62(8):1520. doi: 10.3390/medicina62081520 (PMC13515501; doi:10.3390/medicina62081520)
Supplement: Supplementary file 1 [file medicina-62-01520-s001.zip › medicina-4389143-supplementary.pdf]

Supplementary Materials

Supplementary Table S1. Longitudinal Kaplan–Meier survival parameters stratified by cohort-median psoas muscle index (PMI) at sequential landmarks.

Time Point	Cut-off (cm2/m2)	n (High/ Low)	Median OS - High (mo)	Median OS - Low (mo)	p-Value (OS)	Median TTF - High (mo)	Median TTF - Low (mo)	p-Value (TTF)
PMI_0 (Baseline)	1.97	25/24	17.6	7.4	0.484 (NS)	6.4	5.9	0.768 (NS)
PMI_3 (3-Month)	1.68	25/24	17.6	7.4	0.811 (NS)	6.4	5.9	0.662 (NS)
PMI_6 (6-Month)	1.21	21/21	30.9	5.8	<0.001 (Descriptive)	12.8	4.3	0.003 (Descriptive)

Abbreviations: PMI, psoas muscle index; OS, overall survival; TTF, time-to-treatment failure; NS, not significant. Note: Cut-off thresholds reflect the specific cohort medians computed at each clinical interval. Data for PMI_6 were available for 42 out of 49 patients due to attrition. **p < 0.01; ***p < 0.001.

Methodological Caveat on Landmark Analysis: **Survival estimations relative to the 6-month assessment interval (PMI_6) must be evaluated purely as descriptive observations tracking skeletal muscle depletion in survivors. Because these retrospective strata inherently exclude early dropouts or deaths occurring prior to the 6-month threshold, these specific calculations are highly susceptible to survivor selection artifacts (immortal time bias). Consequently, the nominal p-values derived at this late landmark do not possess independent upfront prognostic validity and should be interpreted with caution.**

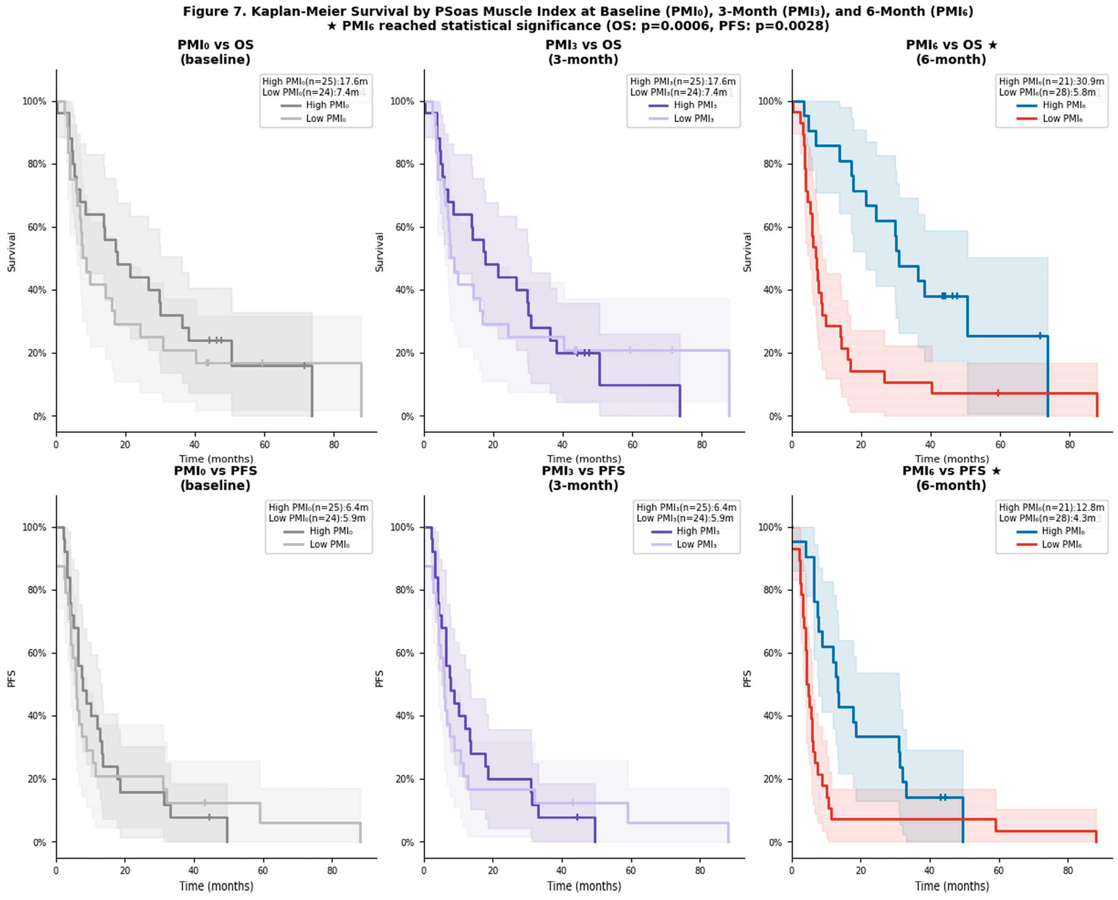

Figure S1. Cumulative Kaplan–Meier curves for overall survival (upper panels) and time-to-treatment failure (lower panels) stratified by time-dependent psoas muscle index (PMI) thresholds. Patient categorization was implemented based on calculated cohort medians at baseline (PMI_0 = 1.97 cm^2/m^2), month 3 (PMI_3 = 1.68 cm^2/m^2), and month 6 (PMI_6 = 1.21 cm^2/m^2). While the 6-month landmark mathematically demonstrated nominal significance for both endpoints, this statistical shift represents a well-documented selection artifact related to survivor bias (immortal time bias) rather than baseline predictive capability. Censored patients are denoted via vertical ticks.

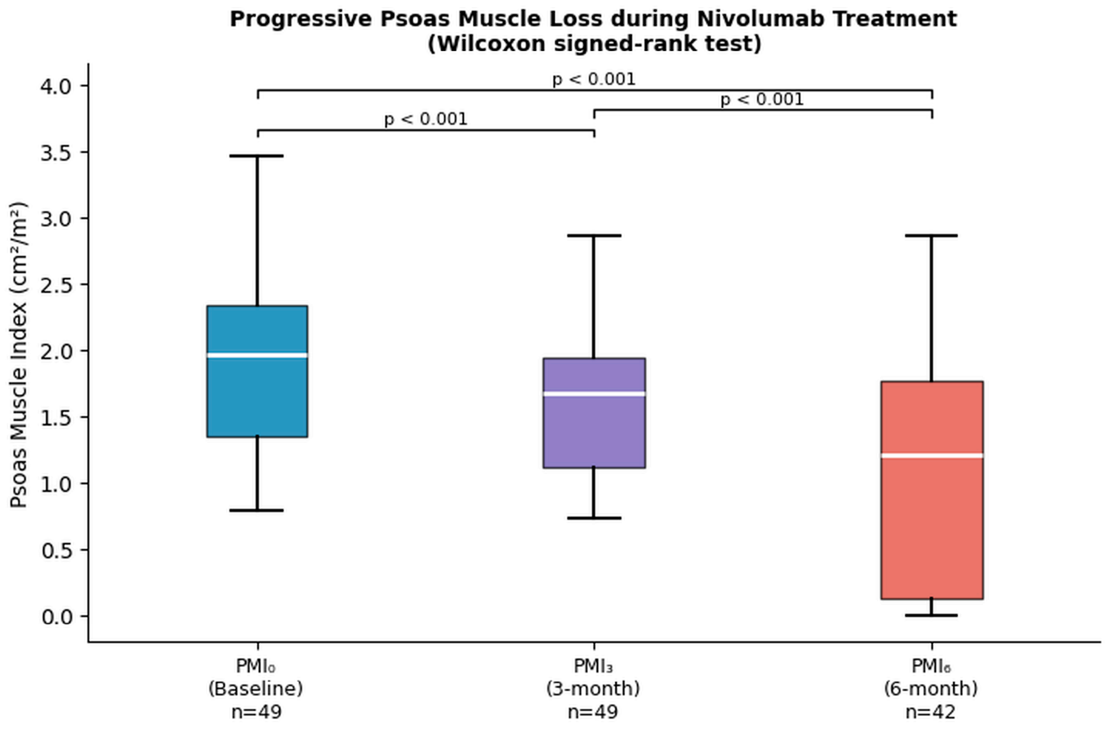

Figure S2. Box plots showing progressive psoas muscle wasting during nivolumab treatment. Box plots track the continuous reduction in psoas muscle index (PMI) values evaluated across predefined observation milestones: baseline (PMI_0, n = 49), month 3 (PMI_3, n = 49), and month 6 (PMI_6, n = 42). Inter-interval comparisons calculated via the Wilcoxon signed-rank test confirmed a systematic, statistically significant degradation pattern over time (all paired transitions, $p < 0.001$). PMI_6 data are descriptive only (survivor selection bias).

Figure S3. Representative CT Image Illustrating the Caliper-Based Ellipsoid Method for PMI_0 Measurement

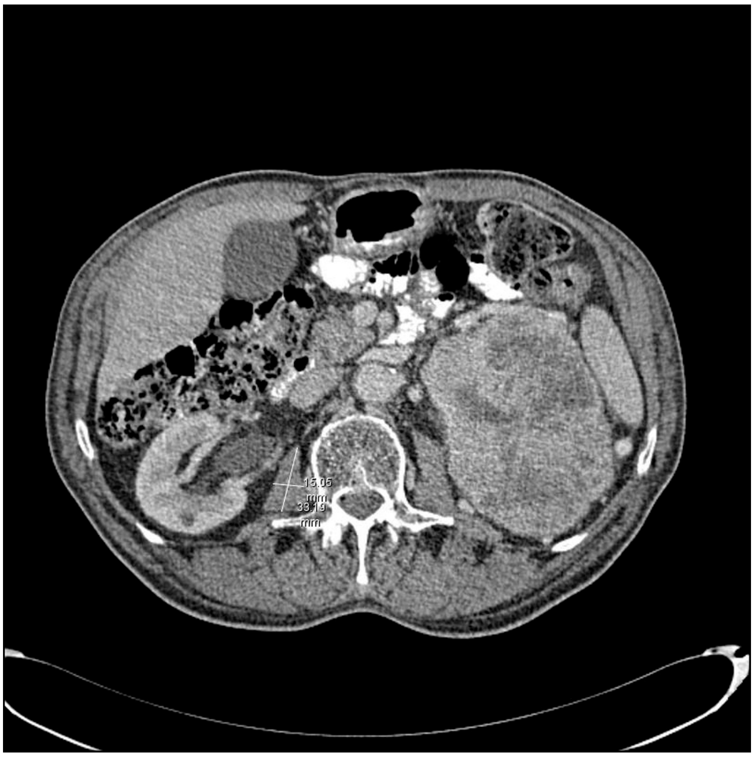

Figure S3. Axial contrast-enhanced computed tomography (CT) image at the third lumbar vertebra (L3) level illustrating the caliper-based ellipsoid measurement of the right psoas muscle cross-sectional area in a representative 66-year-old male patient with left renal cell carcinoma (visible as the large left renal mass). Bilateral anteroposterior (AP) and transverse diameters of the psoas muscle were recorded using the built-in PACS caliper tool (window width 400 HU, level 50 HU). Cross-sectional area was estimated as:
Area (cm^2) = (π/4) × AP diameter × transverse diameter. Illustrated values: right psoas AP = 15.05 mm, transverse = 33.19 mm (estimated area ≈ 3.93 cm^2). The psoas muscle index (PMI_0) was derived as the sum of bilateral psoas areas divided by height squared (cm^2/m^2). Intraclass correlation coefficient (ICC) for the caliper method was 0.94 (95% CI: 0.89–0.97), confirming high inter-observer reproducibility. All patient-identifying information has been anonymised.

Supplementary Table S2. STROBE Statement Checklist for Cohort Studies

This study was reported in accordance with the STROBE (Strengthening the Reporting of Observational Studies in Epidemiology) guidelines for cohort studies. Item-by-item page references are provided below.

STROBE Item	No.	Location in Manuscript
**Title and abstract**	**1**	**Title; Abstract**
**Background/rationale**	**2**	**Introduction**
**Objectives**	**3**	**Introduction (final paragraph)**
**Study design**	**4**	**Methods 2.1**
**Setting**	**5**	**Methods 2.1**
**Participants**	**6**	**Methods 2.1, 2.2**
**Variables**	**7**	**Methods 2.3, 2.4**
**Data sources/measurement**	**8**	**Methods 2.3, 2.4, 2.5**
**Bias**	**9**	**Methods 2.6; Discussion (Limitations)**
**Study size**	**10**	**Methods §2.6 (no a priori power calculation performed; N = 49 with 42 OS events; study powered only to detect HR ≥2.0 at 80% power; exploratory design explicitly stated)**
**Quantitative variables**	**11**	**Methods 2.4, 2.6**
**Statistical methods**	**12**	**Methods 2.6**
**Participants (results)**	**13**	**Results 3.1**
**Descriptive data**	**14**	**Results 3.1; Table 1**
**Outcome data**	**15**	**Results 3.2–3.10**
**Main results**	**16**	**Results 3.3–3.10; Tables 2–6**
**Other analyses**	**17**	**Results 3.7–3.10; Supplementary**
**Key results**	**18**	**Discussion (first paragraph)**
**Limitations**	**19**	**Discussion (Limitations)**
**Interpretation**	**20**	**Discussion; Conclusions**
**Generalisability**	**21**	**Discussion (Limitations)**
**Funding**	**22**	**Funding statement**

Supplementary Figure S4. STROBE-Compliant Patient Flow Diagram

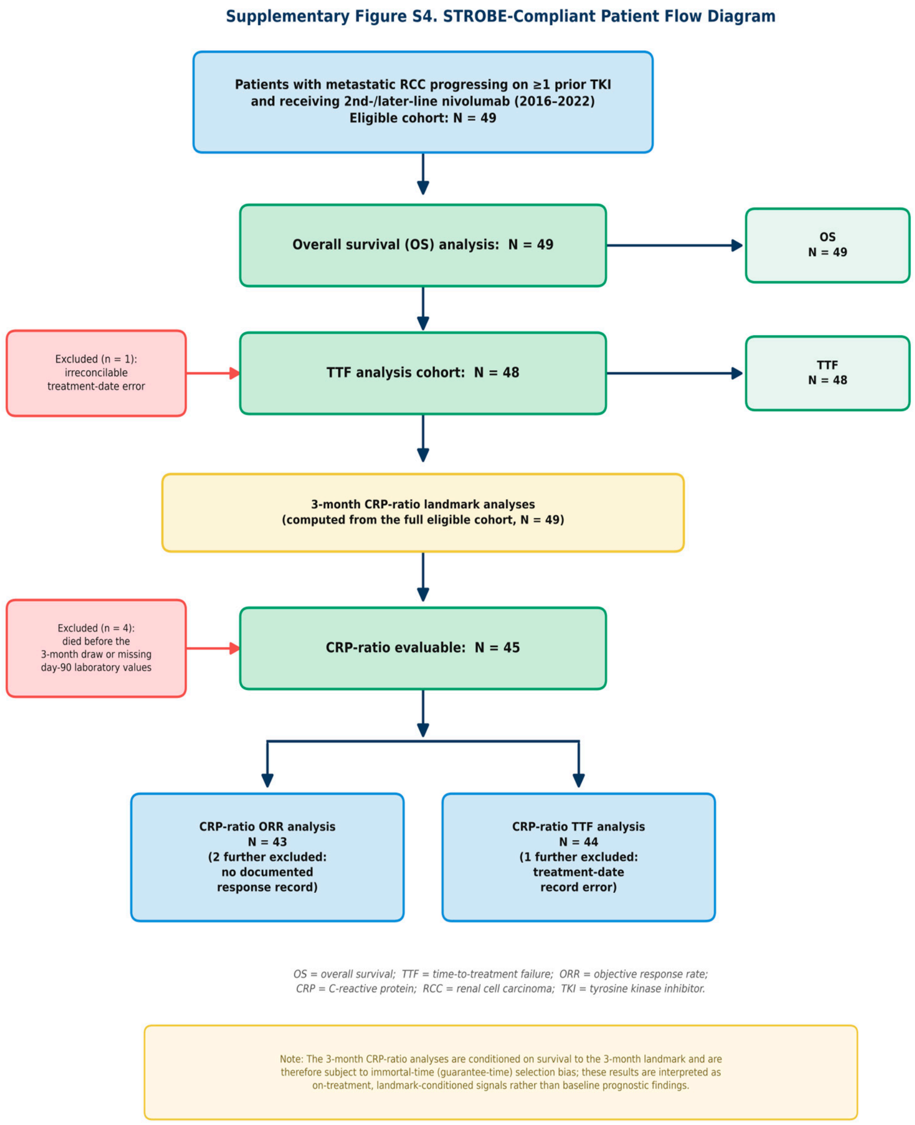

Figure S4. Flow of patients through the study, showing the evaluable population and exclusions at each analytical step. Of 49 consecutive patients with metastatic renal cell carcinoma receiving second- or later-line nivolumab, all 49 were evaluable for overall survival (OS). One patient with an irreconcilable treatment-date error was excluded, leaving 48 for the time-to-treatment failure (TTF) analysis. The 3-month CRP-ratio landmark analyses were computed from the full eligible cohort: 4 patients who died before the 3-month blood draw or lacked day-90 laboratory values were excluded, leaving 45 CRP-ratio-evaluable patients. Of these, 43 had a documented best response and formed the objective response rate (ORR) analysis (2 lacked a response record), and 44 formed the CRP-ratio TTF analysis (1 further excluded for the treatment-date error). The 3-month CRP-ratio analyses are conditioned on survival to the 3-month landmark and are therefore subject to immortal-time (guarantee-time) selection bias.

Supplementary Note S1. Extended Methodological Details for Psoas Muscle Index Measurement

The ellipsoid calliper method may systematically underestimate absolute psoas cross-sectional area compared with manual or automated slice segmentation software. However, the high inter-observer reproducibility observed in our cohort (ICC = 0.94; 95% CI: 0.89–0.97) indicates that within-cohort patient-to-patient ranking remains stable, which is what the cohort-median binary split relies upon; the split is therefore biologically meaningful for the comparative prognostic analyses reported here [10, 24, 25].

No validated Turkish- or Asian-specific PMI thresholds exist for the second- or later-line mRCC context. Notably, our cohort median (1.97 cm^2/m^2) fell below even Asian-derived sarcopenia cut-offs (e.g. Ueki et al. [10]). We therefore deliberately adopted the within-cohort median for relative stratification rather than applying any external absolute threshold. We caution that absolute PMI values — and, consequently, cross-population sarcopenia-prevalence comparisons — are unreliable when measurement techniques differ, and that future investigators should avoid cross-referencing absolute PMI cut-offs obtained by differing methods. A representative CT image illustrating the measurement is provided in Supplementary Figure S3.
